# Maternal fish consumption and child neurodevelopment in Nutrition 1 Cohort: Seychelles Child Development Study

**DOI:** 10.1017/S0007114523000375

**Published:** 2023-10-28

**Authors:** Marie C. Conway, Alison J. Yeates, Tanzy M. Love, Daniel Weller, Emeir M. McSorley, Maria S. Mulhern, Maria Wesolowska, Gene E. Watson, Gary J. Myers, Conrad F. Shamlaye, Juliette Henderson, Philip W. Davidson, Edwin van Wijngaarden, J. J. Strain

**Affiliations:** 1 Nutrition Innovation Centre for Food and Health (NICHE), Ulster University, Coleraine, Northern Ireland; 2 School of Medicine and Dentistry, University of Rochester, Rochester, NY, USA; 3 The Ministry of Health, Mahé, Republic of Seychelles

**Keywords:** Pregnancy, Fish consumption, Neurodevelopment, Cognition, Child development

## Abstract

Maternal fish consumption exposes the fetus to beneficial nutrients and potentially adverse neurotoxicants. The current study investigated associations between maternal fish consumption and child neurodevelopmental outcomes. Maternal fish consumption was assessed in the Seychelles Child Development Study Nutrition Cohort 1 (*n* 229) using 4-day food diaries. Neurodevelopment was evaluated at 9 and 30 months, and 5 and 9 years with test batteries assessing twenty-six endpoints and covering multiple neurodevelopmental domains. Analyses used multiple linear regression with adjustment for covariates known to influence child neurodevelopment. This cohort consumed an average of 8 fish meals/week and the total fish intake during pregnancy was 106·8 (sd 61·9) g/d. Among the twenty-six endpoints evaluated in the primary analysis there was one beneficial association. Children whose mothers consumed larger quantities of fish performed marginally better on the Kaufman Brief Intelligence Test (a test of nonverbal intelligence) at age 5 years (*β* 0·003, 95 % CI (0, 0·005)). A secondary analysis dividing fish consumption into tertiles found no significant associations when comparing the highest and lowest consumption groups. In this cohort, where fish consumption is substantially higher than current global recommendations, maternal fish consumption during pregnancy was not beneficially or adversely associated with children’s neurodevelopmental outcomes.

Fish and seafood are dietary staples for many populations worldwide and globally represent a major source of dietary protein^(1)^. The Food and Agriculture Organization of the United Nations (FAO) estimates that aquatic foods account for at least 20 % of average per capita intake of animal protein for 3·3 billion people^(2)^. Fish is also a rich source of nutrients known to be essential for fetal neurodevelopment, in particular long-chain polyunsaturated fatty acids (LCPUFA), iodine and vitamin D^(3)^. The LCPUFA docosahexanoic acid (DHA) is critical for optimal visual and brain development and deficiencies during fetal growth may have lifelong adverse consequences for brain function^(4)^. Women who consume fish throughout pregnancy are more likely to achieve optimal intakes of these essential nutrients^(5)^. A large body of evidence supports the nutritional benefits of fish consumption throughout pregnancy^(6–8)^. However, fish also contains small amounts of methylmercury (MeHg) and public health consumption guidelines have been formulated with the central aim of limiting possible risk from this naturally occurring environmental pollutant.

Public health advice to pregnant women has been variable. In their 2014 Opinion, the European Food Safety Authority concluded that three to four servings of fish/week (equivalent to >450 g or 16 oz/week) has nutritional benefits for neurodevelopment compared with no fish consumption^(9)^. Similar guidance in the USA recommends that pregnant women should consume 8–12 oz (equivalent to approximately 227–340 g) of fish/week^(10–12)^. The UK advice, last updated in 2004, recommends consuming two portions of fish/week (equivalent to ∼280 g or 10 oz./week) with at least one of these being oily (or fatty) fish^(13)^. Each of these guidelines recommends on a precautionary basis that fish with a high MeHg content (such as shark or swordfish) should be limited or avoided altogether. In many countries, fish consumption in women of childbearing age is significantly below the recommended amounts^(14,15)^. Public confusion about the benefits and risks of fish consumption in the USA contributed to some women avoiding fish altogether when pregnant^(16)^. Limiting fish consumption during pregnancy has possible long-term adverse consequences given its nutritional contribution to the diet.

In 2019, an expert panel conducted a systematic review to evaluate the risks and benefits of seafood consumption (excluding sea mammals) during pregnancy^(7)^. That study reported finding no evidence of an upper limit of intake at which adverse neurodevelopmental outcomes were present. The authors emphasised the benefits of consuming adequate amounts of a wide range of seafood for the greatest cognitive benefits to neurodevelopment, as well as the effect of beneficial nutrients to outweigh potential adverse effects of MeHg exposure^(7,8)^. Fish advisories in the USA are based on epidemiological studies of individuals consuming whales (Faroe Islands) and shark (New Zealand) with co-exposure to multiple other neurotoxicants and the precautionary principle^(17)^. However, findings from the multi-cohort Seychelles Child Development Study (SCDS) support the conclusion that the beneficial effects of nutrients in fish outweigh the possible adverse effects of MeHg^(18–22)^. The SCDS has studied a population that consumes on average more than eight fish meals/week, several times higher than global recommendations^(9,11–13,19)^. The population has one of the highest prenatal MeHg exposures from fish consumption ever studied (> 5 ppm measured in maternal hair), consumes fish with MeHg concentrations similar to those in commercial fish in the UK and USA, and does not consume sea mammals^(23)^. The study has followed three independent longitudinal cohorts over 24 years and found no consistent evidence of adverse associations between MeHg exposure and child neurodevelopmental outcomes^(18–21)^. The SCDS has found beneficial associations between maternal LCPUFA status during pregnancy and early childhood neurodevelopment of offspring, with evidence that *n*-3 and *n*-6 PUFA may ameliorate negative outcomes from MeHg, if any are present, at this level of exposure^(20,22)^.

Previous analyses of the SCDS cohorts focused on individual biomarkers of MeHg exposure and LCPUFA status. The aim of the current study is to investigate associations between maternal fish consumption (consumed as a whole food during pregnancy) and children’s neurodevelopmental outcomes at 9 and 30 months, and 5 and 9 years. The advantage of this approach, as advised by the FDA in their 2014 report on net effects^(10)^, is that it allows both the beneficial contributions of nutrients and potential adverse contributions of MeHg to be considered concurrently. Consequently, results should prove more meaningful for formulating accurate public health guidance.

## Subjects and methods

### Population and location

The SCDS is a longitudinal observational study being conducted in the Republic of Seychelles. The primary aim of the study is to investigate the influence of prenatal MeHg exposure from fish consumption during pregnancy on child neurodevelopmental outcomes^(18)^. The Nutrition Cohort 1 (NC1) has the most comprehensive assessment of fish consumption during pregnancy of any SCDS maternal–child cohort to date and additionally comprehensive assessments of the children’s neurodevelopment. In 2001, we enrolled a total of 300 healthy pregnant women^(22)^. A power calculation determined 250 participants were required to detect a five-point difference on the Bayley Scales of Infant Development II (BSID II) (primary outcome) between the low and high MeHg exposure groups^(19)^. Mothers were recruited during their first antenatal appointment (from 14 weeks of gestation) across the Island of Mahé, the main island of Seychelles. Inclusion criteria were over 16 years of age, native-born Seychellois and having a normal, healthy pregnancy.

Among the 300 women recruited to NC1, there were several exclusions owing to miscarriage/abortion (*n* 12), not being pregnant (*n* 4), illness (*n* 1), relocation (*n* 2) and noncompliance (*n* 8). Additionally, forty-four participants had incomplete dietary data and are not included in this analysis (online Supplementary Fig. 1).

### Ethical approval

This study was conducted according to the guidelines laid down in the Declaration of Helsinki, and all procedures involving participants were reviewed and approved by the Seychelles Ethics Board and the Research Subjects Review Board at the University of Rochester. Written informed consent was obtained from all participants.

### Fish intake data

Dietary data were available at 28 weeks gestation for 229 mothers as detailed in Bonham *et al.*^(24)^ Mothers completed a 4-day semi-quantitative food diary for two consecutive weekdays and two weekend days. The food diaries were available in both English and the native Kreol language and dietitians provided mothers with detailed information on how to complete them. Women were asked to record the amount and types of foods and beverages consumed. Diaries were reviewed locally by dietitians within 1 week of completion. Subsequently, nutritionists from Ulster University, Coleraine reviewed them for any errors or omissions and requested clarification from participants. Food diary data were converted to weight in grams and analysed using dietary analysis software (WISP version 2.0; Tinuviel Software, Warrington, UK) allowing for quantitative food and nutrient intakes to be determined. WISP software was updated with recipe and food composition data for foods commonly eaten in Seychelles using a variety of food composition tables including *The Composition of South African Foods*^(25)^ and *The Concise New Zealand Food Composition Tables*^(26)^. The food diaries provide data on the amount (g/d) of a range of fish consumed during pregnancy. Each fish meal (g/d) was categorised into: *fatty fish*, *lean fish*, *crustaceans*, *molluscs* and *fish products and dishes*. Owing to a large number of non-consumers for the categories of *crustaceans*, *molluscs* and *fish products and dishes* in this cohort, these variables were excluded from analysis. Our analysis focused on the variable of fish consumption (g/d), calculated as the sum of *fatty fish* and *lean fish* consumed.

### Developmental assessment

Seychellois maternal child health nurses specially trained at the University of Rochester administered all neurodevelopmental tests. Children completed testing at ages 9 and 30 months, and 5 and 9 years. All tests were translated into Kreol. At 9 and 30 months children completed the BSID II ^(27)^ as described in Davidson *et al.*^(19)^ At age 5-years, the test battery included the following as described by Strain *et al.*^(28)^: Finger Tapping (Dominant and Non-Dominant hand)^(29)^, the Preschool Language Scale (PLS) (Auditory Comprehension, Verbal Ability and Total Language)^(30)^, the Woodcock–Johnson (WJ) Tests of Achievement (Applied Problems and Letter-Word Recognition)^(31)^, the Achenbach Child Behaviour Checklist (CBCL) (Total score)^(32)^ and the Kaufman Brief Intelligence Test (KBIT) (Verbal Knowledge and Matrices)^(33)^. At age 9 years, the Children’s test battery included the following: CBCL^(32)^, Bender Visual Motor Gestalt^(34)^, Conners’ Attention Deficit Hyperactivity Disorder (ADHD) Index^(35)^, Expressive Vocabulary Test (EVT)^(36)^, KBIT (Verbal Knowledge and Matrices)^(33)^, Peabody Picture Vocabulary (PPV) test^(37)^, Stroop^(38)^, Trail Making Time (Part A and B)^(39)^ and the WJ Tests of Achievement (Applied Problems and Letter-Word Recognition)^(31)^.

### Covariates

Consistent with our previous work^(18,20–22)^, multivariable regression analyses controlled for covariates already known to be associated with child neurodevelopment including: maternal age and IQ (KBIT), child sex, birthweight, and age at testing, socio-economic status (the Hollingshead four-factor SES modified for use in Seychelles), family status (the presence of both parents living with the child), and the home environment (the Paediatric Review of Children’s Environmental Support and Stimulation (PROCESS)).

### Statistical analysis

Descriptive analysis was performed, and all data were expressed as mean ± sd, median, interquartile range and minimum and maximum values. The primary analysis was a series of multiple linear regressions where we separately examined associations between total fish consumption on a continuous scale (g/d) and child neurodevelopmental outcomes at each testing time point, while controlling for maternal age and KBIT, child sex, birthweight, and age at testing, family status, socio-economic status and PROCESS. To examine for any nonlinearity in the association of fish intake and endpoints, we conducted a secondary set of analyses using tertiles of fish consumption, with the lowest tertile as the reference group. Owing to the high levels of fish consumption in our cohort, it was not possible to categorise fish intakes with reference to the current FDA advice, above or below the lower cut point of 8 oz/week (equivalent to 32·4 g/d), as only eleven women reported consumption < 8 oz (227 g/week) of seafood, the lower FDA recommendation and three reported no seafood consumption. Therefore, we divided fish consumption into tertiles and examined their relationship with endpoints. Mothers in the lowest tertile consumed up to 74·5 g/d (median 55 g/d; equivalent to 14 oz/week) total fish. Mothers in the middle tertile consumed 74·6–118·6 g/d (median 97·3 g/d; equivalent to 24 oz/week) and mothers in the highest tertile consumed 118·7–413·3 g/d (median 156·6 g/d; equivalent to 39 oz/week). Analysis was performed with R statistical software, and statistical significance in all analyses was considered a two-sided *P* value <0·05.

## Results

A total of *n* 229 mother–child pairs had complete dietary, neurodevelopmental and covariate data available. The average (sd) maternal age was 27·69 (5·88) years. The cohort comprised *n* 116 girls and *n* 113 boys. The average (sd) maternal total fish consumed in this cohort was 106·8 (61·9) g/d measured at 28 weeks’ gestation as shown in Table 1. As different numbers of children completed each cognitive test, the *n* for each model differs and is shown within Table 2, which also displays summary statistics for the child outcomes at each time point.

**Table 1.** Maternal characteristics of Nutrition Cohort 1 (NC1) with maternal fish consumption and any completed outcomes (*n* 229)

Covariates	Mean	sd	Median	IQR	Min, Max
Maternal age (years)	27·7	5·9	27	23, 32	16, 43
Hollingshead SES	33·93	11·01	33	25, 42	13, 63
Maternal KBIT	86·21	14·19	89	74, 97	48, 117
PROCESS	152·14	14·63	153	141, 161	113, 190
Child birth weight (kg)	3·24	0·47	3·25	2·92, 3·56	1·87, 4·45
Total fish consumption (g/d)	106·8	61·7	97·00	61·00, 131·67	0·00, 413·33

IQR, interquartile range; SES, socio-economic status; KBIT, Kaufmann brief intelligence test; PROCESS, Paediatric Review of Children’s Environmental Support and Stimulation.

**Table 2.** Summary statistics for Nutrition Cohort 1 (NC1) child cognitive outcomes at each time point

Time point	*n*	Mean	sd	Min	Max
9 Months (*n* 229)					
Child age (months)	229	9·51	0·48	8·48	12·22
MDI	226	102·91	8·25	72·00	122·00
PDI	225	105·72	10·38	68·00	141·00
30 Months (*n* 228)					
Child age (months)	228	28·32	1·34	23·52	35·68
MDI	228	85·00	9·51	56·00	115·00
PDI	225	89·81	13·79	50·00	123·00
5 Years (*n* 222)					
Child age (years)	222	5·62	0·30	5·14	6·32
FT dominant	222	23·49	5·72	5·40	39·60
FT non-dominant	222	21·30	4·87	8·60	34·80
PLS auditory comprehension	222	55·57	2·73	47·00	60·00
PLS verbal ability	222	63·10	3·25	51·00	68·00
PLS total language	222	118·68	5·39	100·00	128·00
WJ applied problems	222	15·09	4·14	2·00	24·00
WJ letter-word recognition	222	10·95	6·06	1·00	24·00
CBCL	222	59·30	8·68	25·00	77·00
KBIT verbal	222	11·79	2·77	6·00	18·00
KBIT matrices	222	7·73	1·18	2·00	9·00
9 Years (*n* 216)					
Child age (years)	216	9·52	0·09	9·20	9·92
CBCL	215	37·59	19·34	3·00	103·00
Bender visual motor gestalt	214	22·42	6·10	8·00	40·00
ADHD Conners’ index	215	7·66	8·11	0·00	36·00
EVT	214	79·94	11·80	51·00	126·00
KBIT verbal	215	33·80	9·01	10·00	52·00
KBIT matrices	215	24·03	5·97	12·00	39·00
PPV test	213	133·15	27·62	83·00	189·00
Stroop	206	−21·02	8·97	−61·00	1·00
TM Part A	215	66·47	29·20	23·00	246·00
TM Part B	214	157·46	66·32	52·00	361·00
WJ letter-word recognition	212	66·94	16·03	11·00	76·00
WJ applied problems	215	28·97	4·53	22·00	44·00

NC1, Nutrition Cohort 1; MDI, mental developmental index; PDI, psychomotor developmental index; FT, finger tapping; PLS, Preschool Language Scale; WJ, Woodcock–Johnson; CBCL, Child Behaviour Checklist; KBIT, Kaufman Brief Intelligence Test; ADHD, attention-deficient hyperactivity disorder; EVT, Expressive Vocabulary Test; PPV, peabody picture vocabulary; TM, trail making.

The primary analysis using total fish consumption as a continuous variable and its association with child neurodevelopmental endpoints at each time point is presented in Table 3. Total fish consumption was positively associated with the KBIT Matrices score, a measure of non-verbal intelligence at age 5 years (*β* = 0·003, 95 % CI (0·000, 0·005), *P* = 0·03). There were no adverse associations with child neurodevelopmental outcomes. However, if we had applied the Bonferroni correction for multiple testing and set *P* values at less than 0·002 as statistically significant, then no associations would have met that conservative threshold in primary analysis.

**Table 3.** Associations between maternal fish consumption (continuous) and child cognitive outcomes at each time point adjusted for maternal age and KBIT, child sex, birthweight, and age at testing, family status, socio-economic status and PROCESS

Time point	*n*	Total fish (g/d)			
		*β* effect estimate	*P* value	95 % CI	
				LL	UL
9 Months					
MDI	226	0·000	0·986	−0·017	0·017
PDI	225	0·005	0·645	−0·017	0·027
30 Months					
MDI	228	0·006	0·556	−0·013	0·025
PDI	225	−0·001	0·934	−0·029	0·026
5 Years					
KBIT verbal knowledge	222	0·001	0·665	−0·004	0·007
KBIT matrices	222	**0·003**	**0·030**	**0·000**	**0·005**
PLS auditory comprehension	222	0·001	0·658	−0·004	0·006
PLS verbal ability	222	0·005	0·133	−0·002	0·012
PLS total language	222	0·006	0·246	−0·004	0·017
WJ applied problems	222	0·003	0·384	−0·004	0·011
WJ letter-word recognition	222	0·009	0·070	−0·001	0·019
CBCL	222	−0·002	0·837	−0·020	0·016
FT dominant	222	0·000	0·968	−0·012	0·011
FT non-dominant	222	0·000	0·957	−0·011	0·010
9 Years					
KBIT verbal knowledge	215	−0·012	0·241	−0·032	0·008
KBIT matrices	215	0·005	0·446	−0·008	0·018
EVT	214	0·006	0·609	−0·018	0·031
PPV test	213	0·021	0·496	−0·039	0·080
WJ applied problems	215	0·004	0·398	−0·006	0·014
WJ letter-word recognition	212	0·012	0·484	−0·022	0·047
CBCL	215	0·022	0·296	−0·019	0·063
Bender visual motor gestalt	214	−0·010	0·134	−0·023	0·003
TM Part A	215	0·004	0·904	−0·059	0·067
TM Part B	214	−0·083	0·255	−0·227	0·061
ADHD Conners’ index	216	0·001	0·866	−0·015	0·018
Stroop	206	−0·005	0·608	−0·025	0·015

PROCESS, Paediatric Review of Children’s Environmental Support and Stimulation; MDI, mental developmental index; PDI, psychomotor developmental index; FT, finger tapping; PLS, Preschool Language Scale; WJ, Woodcock–Johnson; CBCL: Child Behaviour Checklist; KBIT: Kaufman Brief Intelligence Test; ADHD, attention-deficient hyperactivity disorder; EVT, Expressive Vocabulary Test; PPV, peabody picture vocabulary; TM, trail making.

Multiple regression models were fit separately and adjusted for maternal age at birth, child age at testing, child sex, birthweight, socio-economic status, family status, home environment and maternal IQ.

A secondary analysis examined fish consumption using tertiles (see Table 4). Among the fifty-two comparisons, there were no significant associations between the highest and the lowest tertiles. At age 5 years, children of mothers in the middle tertile showed a statistically significant adverse difference in score on the WJ Applied Problems scores (a test of mathematical reasoning) from mothers in the lowest tertile. Scores were 1·16 points lower on average (95 % CI (−2·309, −0·007)) than those of mothers in the lowest tertile (*P* = 0·049). We consider this a spurious finding because it was one of fifty-two comparisons, and there was no association between the highest and lowest tertile on this test. In all models, reported associations did not meaningfully change when comparing the associations from models controlling for covariates to those from unadjusted models (see Supplementary Tables). No associations would have been statistically significant if Bonferroni correction for multiple testing and a resultant *P*-value threshold of < 0·002 used.

**Table 4.** Associations between maternal total fish consumption (tertiles of intake) and child neurodevelopmental outcomes at each time point adjusted for maternal age and KBIT, child sex, birthweight, and age at testing, family status, socio-economic status and PROCESS

Time point	*n*	Middle *v*. low tertile*				High *v*. low tertile*			
		*β* effect estimate	95 % CI		*P* value	*β* effect estimate	95 % CI		*P* value
			LL	UL			LL	UL	
9 Months									
MDI	226	0·674	−1·857	3·205	0·600	−0·089	−2·615	2·437	0·945
PDI	225	1·947	−1·355	5·249	0·246	1·741	−1·538	5·020	0·297
30 Months									
MDI	228	1·054	−1·845	3·953	0·474	0·920	−1·959	3·799	0·529
PDI	225	1·148	−3·057	5·353	0·591	−1·160	−5·300	2·981	0·582
5 Years									
KBIT verbal knowledge	222	0·189	−0·683	1·061	0·670	0·004	−0·852	0·859	0·994
KBIT matrices	222	−0·075	−0·457	0·308	0·701	0·151	−0·224	0·526	0·428
PLS auditory comprehension	222	−0·057	−0·879	0·766	0·892	−0·175	−0·981	0·632	0·670
PLS verbal ability	222	0·224	−0·810	1·258	0·670	0·235	−0·779	1·248	0·649
PLS total language	222	0·167	−1·486	1·821	0·842	0·060	−1·561	1·682	0·942
WJ applied problems	222	**−1**·**158**	**−2**·**309**	**−0**·**007**	**0**·**049**	−0·040	−1·169	1·089	0·945
WJ letter-word recognition	222	0·686	−0·868	2·241	0·385	0·775	−0·750	2·300	0·317
CBCL	222	0·813	−1·943	3·570	0·561	−0·119	−2·823	2·584	0·931
FT dominant	222	1·130	−0·689	2·949	0·222	0·553	−1·231	2·336	0·542
FT non-dominant	222	0·614	−1·018	2·246	0·459	0·216	−1·385	1·817	0·790
9 Years									
KBIT verbal knowledge	215	0·302	−2·749	3·353	0·845	−2·363	−5·394	0·667	0·126
KBIT matrices	215	0·048	−1·962	2·057	0·963	−0·005	−2·001	1·991	0·996
EVT	214	0·919	−2·944	4·782	0·640	0·072	−3·753	3·896	0·971
PPV test	213	0·993	−8·298	10·284	0·833	2·401	−6·813	11·615	0·608
WJ applied problems	215	0·608	−0·886	2·102	0·423	0·479	−1·005	1·964	0·525
WJ letter-word recognition	212	2·606	−2·765	7·977	0·340	1·059	−4·309	6·427	0·698
CBCL	215	2·256	−4·043	8·554	0·481	5·613	−0·643	11·869	0·078
Bender visual motor gestalt	214	0·751	−1·252	2·754	0·461	−0·958	−2·950	1·035	0·344
TM Part A	215	−4·741	−14·465	4·983	0·338	2·547	−7·111	12·206	0·604
TM Part B	214	−2·587	−24·908	19·734	0·819	−4·541	−26·814	17·732	0·688
ADHD Conners’ index	216	−0·036	−2·617	2·545	0·978	0·980	−1·594	3·553	0·454
Stroop	206	1·363	−1·674	4·400	0·377	0·399	−2·656	3·454	0·797

PROCESS, Paediatric Review of Children’s Environmental Support and Stimulation; MDI, mental developmental index; PDI, psychomotor developmental index; FT, finger tapping; PLS, Preschool Language Scale; WJ, Woodcock–Johnson; CBCL, Child Behaviour Checklist; KBIT, Kaufman Brief Intelligence Test; ADHD, attention-deficient hyperactivity disorder; EVT, Expressive Vocabulary Test; PPV, peabody picture vocabulary; TM, trail making.

*Tertile median g/d (tertile range g/d); range of fish intake for each tertile at each time point is as follows: 9 months: low (*n* 77) = 55·0 g/d (0–74·5), middle (*n* 76) = 97·3 g/d (74·6–118·6), high (*n* 76) = 156·6 g/d (118·7–413·3); 30 months: low (*n* 76) = 55·0 g/d (0–74·3), middle (*n* 76) = 97·3 g/d (74·4–118·8), high (*n* 76) = 156·6 g/d (118·9–413·3); 5 years: low (*n* 74) = 55·0 g/d (0–74·7), middle (*n* 74) = 96·8 g/d (74·8–118·4), high (*n* 74) = 155·3 g/d (118·5–413·3); 9 years: low (=72) = 55·4 g/d (0–74·3), middle (*n* 72) = 97·6 g/d (74·4–118·8), high (*n* 72) = 155·3 g/d (118·9–413·3).

## Discussion

In the primary analysis examining the association of maternal fish consumption as a continuous variable with the twenty-six neurodevelopmental endpoints, we found one positive association. The children’s KBIT matrices, a test of nonverbal intelligence, at age 5 years improved as fish consumption increased. In a secondary analysis categorising fish consumption by tertiles, we found no significant associations between the highest and lowest tertiles. However, there was a statistically significant adverse difference in score on the WJ Applied Problems scores in children from mothers in the middle tertile when compared with children from mothers in the lowest tertile. We interpret our study as providing no clear evidence in either the primary or secondary analysis of beneficial or adverse associations between maternal fish consumption and children’s neurodevelopment. These results are consistent with our earlier findings in this cohort and findings of two recent systematic reviews which showed no adverse associations of fish consumption.

In our earlier assessment of this cohort, we found the mothers’ total *n*-3 PUFA status (a proxy for fatty fish consumed during pregnancy) was positively associated with the PDI in this age group^(22)^. This finding suggested that higher *n*-3 PUFA may be contributing to the improved psychomotor development of infants at this age. The guidance from fish advisories differs worldwide, but the most common advice during pregnancy is to consume fish 2 to 3 times/week, with at least one portion being fatty fish^(9–12)^. The suggested benefits are believed to be mainly attributable to DHA, a crucial nutrient in pregnancy for brain neurodevelopment^(4)^. The benefits of DHA for neurodevelopment are well established^(4)^, but the evidence for prenatal DHA supplementation remains inconclusive^(40)^.

In contrast, there is convincing evidence of the benefits of fish consumption in pregnancy for infant neurodevelopment from multiple studies that have evaluated fish as a whole food. Two rigorous scientific reviews of the evidence in this field concluded that there were no adverse associations of fish consumption with children’s neurodevelopment^(7,8)^. The reviews evaluated data from forty-four publications where the range of beneficial outcomes included improved visual acuity, early language and communication skills, IQ and social skills in children^(7,8)^. In these studies, fish consumption ranged from ∼4 oz (113 g) per week up to > 100 oz (2835 g or ≥405 g/d) per week^(7,8)^). Women in the SCDS NC1 consumed on average approximately 106 g/d (3·7 oz) fish, which is equivalent to 26 oz/week; these quantities are substantially more than the FDA advice to consume 8 to 12 oz/week in pregnancy.

As the Seychellois are such a high fish-consuming population, exposure to MeHg is several times higher than in the USA or UK. However, it is important to note that MeHg concentrations in fish in the Seychelles^(23)^ are the same as in countries such as USA^(41)^; therefore, it is the high levels of fish consumption, rather than Seychelles fish containing higher MeHg that leads to higher MeHg exposure for the Seychellois population. Our results add further evidence to the existing reports which found no adverse associations with high fish consumption during pregnancy^(7)^. We have previously reported that the nutrients, mainly LCPUFA, present in fish are likely to overcome any potential adverse toxic effects of prenatal MeHg exposure^(20–22)^. Our findings add to the evidence supporting the safety of consuming fish that has only naturally acquired amounts of MeHg.

Strengths of our study include its prospective longitudinal double-blind exposure design and neurodevelopmental evaluations by specially trained nurse evaluators at multiple time points using a comprehensive battery of tests including measures of IQ and verbal development. Also, detailed dietary data collected prospectively through the completion of 4-d food diaries, a method which minimises some of the errors typically associated with interviewer technique and memory recall^(42)^. The dietary data were further strengthened by our update of the WISP dietary analysis software with food composition data for foods specific to Seychelles and extensive review of the data by dietitians in Seychelles and nutritionists at Ulster University. Additionally, in Seychelles, consuming sea mammals is prohibited and there is no co-exposure to other pollutants which could potentially be detrimental to fetal neurodevelopment. Limitations of the study include it being an observational epidemiology study and unmeasured covariates might have been omitted, and the sample size is relatively small.

### Conclusion

In this cohort, where fish consumption is substantially higher than current global recommendations, maternal fish consumption during pregnancy was not beneficially or adversely associated with children’s neurodevelopmental outcomes in primary or secondary analyses across numerous time points up to 9 years of age.

## Supplementary material

For supplementary material accompanying this paper visit https://doi.org/10.1017/S0007114523000375. click here to view supplementary material
