# Supplementary material for: Maternal fish consumption and child neurodevelopment in Nutrition 1 Cohort: Seychelles Child Development Study
Source: Br J Nutr. 2023 Feb 10;130(8):1366–72. doi: 10.1017/S0007114523000375 (PMC10511674; doi:10.1017/S0007114523000375)
Supplement: Supplementary file 1 [file S0007114523000375sup.zip › S0007114523000375sup001.docx]

N=273 eligible mother-child pairs

N=300 mother-child pairs

N=27 exclusions:

Miscarriage/abortion (n=12)

Not being pregnant (n=4)

Illness (n=1)

Relocation (n=2)

Noncompliance (n=8)

N=44 participants had incomplete dietary data and were not included in this analysis

N=229 mother-child pairs included in current analysis

**Supplemental Figure 1: Participant flow chart**
