# Supplementary material for: Maternal fish consumption and child neurodevelopment in Nutrition 1 Cohort: Seychelles Child Development Study
Source: Br J Nutr. 2023 Feb 10;130(8):1366–72. doi: 10.1017/S0007114523000375 (PMC10511674; doi:10.1017/S0007114523000375)
Supplement: Supplementary file 1 [file S0007114523000375sup.zip › S0007114523000375sup002.docx]

| **Supplementary Table 1*.* Associations between maternal fish consumption (continuous) and child cognitive outcomes at each time point unadjusted. Models were fit separately.** | | | | | |
| --- | --- | --- | --- | --- | --- |
|  |  | **Total fish (g/d)** | | | |
|  |  |  |  | **95% CI (LL, UL)** | |
| **Time point** | ***n*** | **β effect estimate** | **P value** | **LL** | **UL** |
| *9 Months* |  |  |  |  |  |
| MDI | 226 | -0·002 | 0·825 | -0·020 | 0·016 |
| PDI | 225 | 0·004 | 0·736 | -0·018 | 0·026 |
| *30 Months* |  |  |  |  |  |
| MDI | 228 | 0·008 | 0·418 | -0·012 | 0·028 |
| PDI | 225 | 0·004 | 0·775 | -0·025 | 0·033 |
| *5 Year* |  |  |  |  |  |
| KBIT Verbal Knowledge | 222 | 0·002 | 0·598 | -0·004 | 0·007 |
| KBIT Matrices | 222 | **0·003** | **0·033** | **0·000** | **0·005** |
| PLS Auditory Comprehension | 222 | 0·002 | 0·494 | -0·004 | 0·008 |
| PLS Verbal Ability | 222 | 0·006 | 0·097 | -0·001 | 0·013 |
| PLS Total Language | 222 | 0·008 | 0·178 | -0·004 | 0·019 |
| WJ Applied Problems | 222 | 0·005 | 0·302 | -0·004 | 0·013 |
| WJ Letter-Word Recognition | 222 | 0·011 | 0·079 | -0·001 | 0·024 |
| CBCL | 222 | -0·001 | 0·875 | -0·020 | 0·017 |
| FT Dominant | 222 | 0·000 | 0·941 | -0·012 | 0·013 |
| FT Non-Dominant | 222 | 0·000 | 0·974 | -0·011 | 0·010 |
| *9 Year* |  |  |  |  |  |
| KBIT Verbal Knowledge | 215 | -0·012 | 0·213 | -0·032 | 0·007 |
| KBIT Matrices | 215 | 0·005 | 0·471 | -0·008 | 0·018 |
| EVT | 214 | 0·008 | 0·515 | -0·017 | 0·034 |
| PPV Test | 213 | 0·017 | 0·584 | -0·043 | 0·077 |
| WJ Applied Problems | 215 | 0·004 | 0·429 | -0·006 | 0·014 |
| WJ Letter-Word Recognition | 212 | 0·011 | 0·521 | -0·024 | 0·046 |
| CBCL | 215 | 0·019 | 0·368 | -0·023 | 0·061 |
| Bender Visual Motor Gestalt | 214 | -0·009 | 0·195 | -0·022 | 0·005 |
| TM Part A | 215 | 0·003 | 0·931 | -0·061 | 0·066 |
| TM Part B | 214 | -0·078 | 0·287 | -0·222 | 0·066 |
| ADHD Conners' Index | 216 | 0·002 | 0·831 | -0·016 | 0·020 |
| Stroop | 206 | -0·004 | 0·709 | -0·024 | 0·016 |
| MDI: Mental Developmental Index; PDI: Psychomotor Developmental Index; FT: Finger-Tapping; PLS: Preschool Language Scale; WJ: Woodcock Johnson; CBCL: Child Behavior Checklist; KBIT: Kaufman Brief Intelligence Test; ADHA: Attention Deficient Hyperactivity Disorder; EVT: Expressive Vocabulary Test, PPV: Peabody Picture Vocabulary, TM: Trail Making | | | | | |

| **Supplementary Table 2*.* Associations between maternal total fish consumption (tertiles of intake) and child neurodevelopmental outcomes at each time point unadjusted. Models were fit separately.** | | | | | | | | | | |
| --- | --- | --- | --- | --- | --- | --- | --- | --- | --- | --- |
|  |  | **Middle vs Low Tertile*** | | | | **High vs Low Tertile*** | | | | |
|  |  |  | **95% CI (LL, UL)** | |  | |  | **95% CI (LL, UL)** | |  |
| **Time point** | ***n*** | **β effect estimate** | **LL** | **UL** | **P value** | | **β effect estimate** | **LL** | **UL** | **P value** |
| *9 Months* |  |  |  |  |  | |  |  |  |  |
| MDI | 226 | 0·222 | -2·442 | 2·885 | 0·870 | | -0·739 | -3·403 | 1·925 | 0·585 |
| PDI | 225 | 2·263 | -1·088 | 5·614 | 0·185 | | 1·547 | -1·782 | 4·876 | 0·361 |
| *30 Months* |  |  |  | |  | |  |  |  |  |
| MDI | 228 | 1·842 | -1·202 | 4·886 | 0·234 | | 0·868 | -2·176 | 3·913 | 0·575 |
| PDI | 225 | 2·732 | -1·714 | 7·178 | 0·227 | | -0·739 | -5·156 | 3·678 | 0·742 |
| *5 Year* |  |  |  | |  | |  |  |  |  |
| KBIT Verbal Knowledge | 222 | 0·338 | -0·563 | 1·239 | 0·461 | | 0·000 | -0·901 | 0·901 | 1·000 |
| KBIT Matrices | 222 | -0·027 | -0·409 | 0·355 | 0·889 | | 0·149 | -0·233 | 0·531 | 0·444 |
| PLS Auditory Comprehension | 222 | 0·108 | -0·781 | 0·998 | 0·811 | | -0·135 | -1·025 | 0·754 | 0·765 |
| PLS Verbal Ability | 222 | 0·568 | -0·488 | 1·623 | 0·290 | | 0·311 | -0·745 | 1·366 | 0·562 |
| PLS Total Language | 222 | 0·676 | -1·075 | 2·427 | 0·448 | | 0·176 | -1·575 | 1·927 | 0·843 |
| WJ Applied Problems | 222 | -0·689 | -2·034 | 0·655 | 0·314 | | 0·014 | -1·331 | 1·358 | 0·984 |
| WJ Letter-Word Recognition | 222 | 1·446 | -0·517 | 3·409 | 0·148 | | 0·892 | -1·071 | 2·855 | 0·371 |
| CBCL | 222 | 0·770 | -2·051 | 3·591 | 0·591 | | -0·041 | -2·862 | 2·780 | 0·977 |
| FT Dominant | 222 | 0·616 | -1·244 | 2·476 | 0·514 | | 0·795 | -1·065 | 2·654 | 0·401 |
| FT Non-Dominant | 222 | 0·332 | -1·250 | 1·915 | 0·679 | | 0·257 | -1·326 | 1·840 | 0·750 |
| *9 Year* |  |  |  | |  | |  |  |  |  |
| KBIT Verbal Knowledge | 215 | 0·584 | -2·365 | 3·533 | 0·697 | | -2·528 | -5·466 | 0·411 | 0·091 |
| KBIT Matrices | 215 | 0·254 | -1·722 | 2·229 | 0·801 | | -0·153 | -2·122 | 1·816 | 0·879 |
| EVT | 214 | 1·158 | -2·761 | 5·077 | 0·561 | | 0·111 | -3·780 | 4·002 | 0·955 |
| PPV Test | 213 | -0·200 | -9·381 | 8·982 | 0·966 | | 0·606 | -8·543 | 9·754 | 0·896 |
| WJ Applied Problems | 215 | 0·517 | -0·981 | 2·016 | 0·497 | | 0·222 | -1·271 | 1·715 | 0·770 |
| WJ Letter-Word Recognition | 212 | 3·346 | -1·961 | 8·654 | 0·215 | | 0·632 | -4·675 | 5·940 | 0·815 |
| CBCL | 215 | 2·436 | -3·928 | 8·801 | 0·451 | | 5·361 | -0·981 | 11·703 | 0·097 |
| Bender Visual Motor Gestalt | 214 | 0·676 | -1·340 | 2·692 | 0·509 | | -0·812 | -2·821 | 1·197 | 0·426 |
| TM Part A | 215 | -4·258 | -13·872 | 5·357 | 0·384 | | 3·486 | -6·095 | 13·067 | 0·474 |
| TM Part B | 214 | -2·483 | -24·448 | 19·482 | 0·824 | | -1·230 | -23·195 | 20·736 | 0·912 |
| ADHD Conners' Index | 216 | -0·519 | -3·195 | 2·156 | 0·702 | | 1·300 | -1·375 | 3·976 | 0·339 |
| Stroop | 206 | 0·798 | -2·226 | 3·822 | 0·603 | | 0·397 | -2·648 | 3·443 | 0·797 |
| MDI: Mental Developmental Index; PDI: Psychomotor Developmental Index; FT: Finger-Tapping; PLS: Preschool Language Scale ; WJ: Woodcock Johnson; CBCL: Child Behavior Checklist; KBIT: Kaufman Brief Intelligence Test; ADHA: Attention Deficient Hyperactivity Disorder; EVT: Expressive Vocabulary Test, PPV: Peabody Picture Vocabulary, TM: Trail Making; *tertile median g/day (tertile range g/d) fish intake for each tertile at each time point is as follows: 9 months: low (n=77) = 55g/day (0- 74·5), medium (n=76) = 97·25g/d (74·5- 118·6), high (n=76) = 156·58g/d (118·6- 413·3); 30 months: low (n=76) = 55g/d (0- 74·3), medium (n=76) = 97·25g/d (74·3- 118·8), high (n=76) = 156·58g/d (118·8- 413·3); 5 years: low (n=74) = 55g/d (0- 74·7), medium (n=74) = 96·75g/d (74·7- 118·4), high (n=74) = 155·33g/d (118·4-413·3); 9 years: low (=72) = 55·41g/d (0- 74·3), medium (n=72) = 97·58g/d (74·3- 118·8), high (n=72) = 155·33g/d (118·8- 413·3). | | | | | | | | | | |
